# Enhancing performance measurement of public procurement processes through the application of procurement delay index

**DOI:** 10.1016/j.heliyon.2024.e25672

**Published:** 2024-02-06

**Authors:** Jemima A. Ottou, Bernard K. Baiden, Gabriel Nani, Martin Morgan Tuuli

**Affiliations:** a Department of Management Science, Business School, Ghana Institute of Management and Public Administration (GIMPA), Accra, Ghana; b Department of Construction Technology and Management, Kwame Nkrumah University of Science and Technology, Kumasi, Ghana

**Keywords:** Delay, Performance measurement, Procurement delay index, Timeliness

## Abstract

Considering procurement process performance assessment, measurement systems such as balance scorecard and capability maturity models are used to assess procurement performance to give a reasonable indication of actual performance. These tools are ideal for practical applications that depend on other performance criteria but are short of comparing planned to actual timelines set for procurement processes. This fosters persistent delays in public procurement. Six Sigma implementation benefits including addressing process delays have fostered its implementation for service improvements. Unfortunately, Six Sigma implementation in public procurement is very rare largely due to its expensive nature. The objective of this study is to develop a Procurement Delay Index (PD*index*): a performance measure in the context of a Six Sigma methodology that provides a specific value to describe the delays in the procurement process. To enhance uniformity in performance measurement of process timeliness, a rating scale for determining the timeliness of the procurement process is proposed. A practical demonstration of the application of PD*index* for use by procurement professionals is also presented. PD*index* serves as a standard by which to determine procurement processes’ timeliness and is less expensive to implement. A PD*index* of 3σ has been recommended as the acceptable limit for procurement process delays.

## 1 Introduction

Since Motorola introduced Six Sigma as a performance improvement strategy in manufacturing in the mid-1980s, the concept has been embraced by all sectors. Over three decades now, Six Sigma application benefits have encouraged its application in diverse areas including manufacturing [1,2], service [3,4], and construction [5,6]. However, its application in procurement is rare with sparse research on it [7,4]. Meanwhile, procurement management has made impressive progress with a recent focus on sustainable procurement [8,3]. Despite this, delay in public procurement processes is yet to be fully explored in literature and practice. Thus, delays in public procurement processes persist, leading to cost overruns due to poor economic conditions [9].

To address these delays and enhance the performance of supply chains, entities are expected to plan their procurement processes appropriately and implement procurement activities closely with the plans, especially in terms of timelines. Unfortunately, countries such as the Czech Republic, the United Kingdom [10], and Ghana have recorded undue delays in their public procurement processes despite proper procurement planning. In Ghana, public entities that are benchmarks in procurement management have recorded undue delays in their procurement processes [11]. The reason is that the performance criteria used in assessing benchmark entities do not accurately assess process delays. Since the main thrust of Six Sigma is its measurement metrics [5,12], it is a promising tool for addressing the flaws in procurement performance measurement.

Attempts to encourage Six Sigma application in procurement are limited because of the anticipated implementation challenges [13,2]. Despite these challenges, several researchers [13,3,4,6] have advocated its implementation due to the associated benefits [14]. Thus, there have been advocacies to focus on the application of the Six Sigma metric to measure performance [13,15,16,17]. For instance Ref. [18], noted that the traditional capability indices employed in Six Sigma methodology indicates a range of quality level and not a specific value. Thus [19], developed the Taguchi Six Sigma quality index to measure process performance. Following this line of thought, Six Sigma metrics can used to measure delays in procurement processes. This can serve as a subtle and cheaper means of encouraging the implementation of Six Sigma in procurement processes.

In this paper, the concept of Six Sigma and the statistical basis for sigma level is discussed leading to the development of PD*index*. Next, the 2014 Ghana public procurement assessment criteria are reviewed to highlight the little attention given to the assessment of delays. Then the PD*index* is validated by demonstrating its application within a case study. The purpose of this study is as follows:

i. To develop the procurement delay index (PD*index*) measured in sigma (σ) as a performance measure that provides a specific value to describe procurement process delays. The PD*index* is expected to complement existing performance measures to accurately ascertain the actual performance of a procurement process by measuring the delay rate.
ii. To propose a rating scale for determining the timeliness of procurement processes. This will encourage a focus on timeliness, facilitate the implementation of Six Sigma in procurement processes, and improve the entire supply chain.

## 2 Related work

### 2.1 The concept of six sigma

Six Sigma is a disciplined project-oriented statistically-based approach for reducing process variability by eliminating defects from processes [13]. From a statistical perspective, Six Sigma is a metric of process measurement symbolized by the Greek letter (σ) which represents the amount of variation within a normal data distribution. It is a data-driven process that applies scientific analysis to relate quality level to 3.4 defects per million opportunities (DPMO) assuming a 1.5 shift of process average [13,20]. This provides a robust advantage in improving quality in processes because it allows for designs that are comparatively resistant to natural or inevitable sources of variation [21]. Table 1 illustrates the defect rate per million opportunities in different sigma levels.

**Table 1:** Relationship between sigma level and defects per million opportunities.

Sigma (σ) level	Defects per million opportunities
0	1,000,000
1	690,000
2	308,538
3	66,807
4	6210
5	233
6	3.4

Source: Extracted from [22].

Six Sigma is based on a statistical distribution called the standard normal distribution. This distribution is usually illustrated by a symmetrical bell-shaped curve. The curve denotes the total population with an infinite series of segments in both directions. Each segment is a deviation from the mean and is called the Sigma (σ) in statistics. As the curve contracts, the cumulative population around the mean and within the specification limits represents excellent performance. This can be used to establish the capability of any process. Process capability is the ability of a process to meet its design specification which is expressed in using a process capability index. This index simply compares the design specification to actual performance to establish whether actual performance is well within the boundaries of expected performance. In establishing the design specification in practice, the control chart by Walter Shewhart is used to set the upper and lower specification limits within the 3 sigma level. This refers to 3 standard deviations from the process mean. The reason is that 3 Sigma refers to processes that operate efficiently to produce items of the highest quality in practice. This thinking is based on Shewhart's theory that even perfectly designed processes exhibit a certain amount of variability in output measurements. Thus, the capability index can also be expressed as an analogous sigma level or DPMO estimate.

In practice, it is better for a sigma level or DPMO estimate to focus on one performance indicator as applying it to several performance indicators to ascertain the overall defect rate may be fraught with error. This is because some performance indicators may carry heavier weight than others. However, this single indicator should be related to a process that can be upraised by the customer. Process cycle time is a common focus in this regard. The ideal expected level of performance should however be defined.

Even though the Six Sigma level for process performance is ideal, achieving this level is not practical. Hence, the standard practice is for processes to fall within three standard deviations (3σ) representing 99.7% of processes operating within the design specification limits [23]. This requires that data on the actual process is collected and compared to the design specification to measure performance. It is necessary to note that government performance is largely influenced by the performance of public procurement.

### 2.2 Public procurement and government performance

Governments rely heavily on public procurement to acquire goods and services. Public procurement is a process used by public entities to acquire goods, works, and services with public funds [24]. As of November 25, 2021, the Organisation for Economic Co-operation and Development (OECD) stated on its website that the total volume of public procurement accounts for 12% of GDP in its countries. The situation is similar in Ghana. As of September 5, 2018, EUROPEANCEO stated on its website that between 50% and 70% of Ghana's budget is spent on procurement representing 14% of its GDP. Thus, there is a need to constantly improve the performance of public procurement to prudently utilize limited resources. However, ascertaining the level of performance requires constant performance measurement. Thus, Ghana's public procurement processes were rigorously assessed in 2014. A review of this procurement assessment exercise will reveal the pertinent aspect of procurement that requires attention.

### 2.3 Ghana public procurement assessment exercise

Ghana boasts of a well-structured public procurement system which is expected to yield accurate procurement performance measurement. The [25] and the [26] provide guidelines for public procurement in Ghana. These acts also standardize procurement practices and establish the public procurement regulatory authority, known as the Public Procurement Authority (PPA). The PPA is responsible for the effective implementation of the Public Procurement Law in Ghana. PPA seeks to ensure fairness, transparency, and non-discrimination in public procurement. This is required to promote a competitive local industry and increase confidence in public procurement processes.

In fulfillment of its mandate, the PPA undertakes an annual assessment of procurement activities by public entities. In 2014, the PPA assessed all public entities in Ghana. This assessment rated public entities according to the quality of their procurement practices. The results of this assessment served as a performance measure.

The PPA adopted a ‘balanced scorecard’ based performance indicators for the assessment after which they ranked the various entities into some pre-determined maturity levels. This technique follows the norm as several researchers [3,27,28,29] consider the balanced scorecard as the widely used system for measuring the performance of procurement processes. The performance criteria used for the assessment were centered around the existence of prescribed procurement structures; using qualified procurement practitioners; preparation of procurement plans; and ensuring proper functioning of structures and staff in terms of ensuring process transparency, fairness, equity, and accountability.

#### 2.3.1 Details of the assessment method used by the Public Procurement Authority in Ghana

The assessment criteria used by the PPA were based on 22 Key Performance Criteria (KPC) classified under 4 major sections with 59 sub-criteria (see Appendix 1). Each of the sub-criteria attracts some points ranging from 5 to 9. The total points for all the 59 sub-criteria works up to 500 points. After scoring the entities based on the foregoing sub-criteria, weights for the 4 major sections are then applied to points. Weight for section 1: management systems is 0.15; section 2: information and communication are 0.15; section 3: procurement process is 0.45; and section 4: contract management is 0.25. Then the product of the weights and the points for each sub-criteria obtained in the 4 major sections are summed up to arrive at a total weighted score (see Appendix 2). The total weighted score forms the basis for ranking the entities.

The entities are ranked based on some maturity criteria namely, excellent, matured, maturing, conforming, and non-conforming (see Appendix 2). This method was adopted to assess the entities leading to the publication of [30]. This table is the only assessment carried out in Ghana that rated the procurement performance of all public entities and presented it as a published document. A total of 546 public entities were assessed. The assessment revealed that 38, 195, 169, 102, and 42 entities were ranked excellent, matured, maturing, conforming, and non-conforming respectively. The excellent-ranked entities representing 6.96% of all public entities in Ghana are thus the benchmark entities in public procurement. It is therefore expected that benchmark entities should not experience delays in their procurement processes but this is not the case [11]. This revelation led to further review of the performance criteria and methods used to ascertain the performance of these public entities.

A review of the 59 key performance sub-criteria revealed that even though these criteria were very comprehensive and touched on all facets of procurement, the criteria relating to process delays were given a very low rating. The review revealed that out of the 59 key performance sub-criteria, only three namely, 1C4, 3B1, and 3F1 touched on the timeliness of the activities. Table 2 presents details of the three specific assessment criteria that touched on time in the 2014 assessment exercise.

**Table 2:** Details of Delay Assessment method by the Public Procurement Authority.

Criteria	Proof of Evidence	Maximum Point	Measurement for Delays
Criteria 1C: Monitoring and Control Systems: Sub-criteria 1C4- Procurement Entity has defined methods of contract administration responsibilities which include inspection and acceptance procedures and methods to review and issue contract amendments in a timely manner.”	i.Contract document clearly indicates contract management procedures with clear cut responsibilities for review & amendments ii. Approvals by relevant approving Authorities	6 3	None None
Criteria 3 B: Notice: Sub-criteria 3B1 - Publication of notices are mandatory and publicly advertised in a timely manner according to respective procurement methods and thresholds, where applicable	Has Entity Used NCT/ICT Before? (Yes/No) i.Copies of Advertisements (3) ii. Advertisements according to Procurement Plan (3) iii. Advertisements posted on PPA's website (3)	3 3 3	None Timelines are considered and if they are not as planned, the entity scores 0 points None
Criteria 3 F - Tender Opening: Sub-criteria 3F1 - Procurement Entity opens tenders at the same time as the deadline for the receipt of tenders”	i.Specific Dates for Tender Opening – 4 ii. Times for Tender Openings −3 iii. Minutes of Tender Opening −2	4 3 2	Timelines are considered and if they are not as planned, the entity scores 0 points Timelines are considered and if they are not as planned, the entity scores 0 points None

From Table 2 and it can be seen that out of the 500 points for the assessment criteria, only 10 points were allocated to delays. There was no criterion purposely to compare the planned timelines of the various process steps to the actual timelines. Thus, it can be seen that delays were not a major focus of the performance assessment. However, in measuring the performance of procurement, delays cannot be rated low because of their effect on overall performance. To address process delays, procurement activities must be carried out promptly. This requires that time is viewed as an important performance criterion in procurement.

### 2.4 Time as an important performance criterion in public procurement

Time is widely used as a performance measure for projects [31] thus the use of terms such as process cycle time, lead time, and on-time delivery. Time wasted can never be regained hence the need to closely monitor time spent on processes in businesses. There is a statistically significant correlation between time and cost [32] hence, the adage *‘time is money’*. This correlation can be so strong that cost information can be the sole basis for estimating construction project time. Timeliness must therefore be viewed as a critical performance measure in procurement processes to accurately ascertain performance. Timeliness used here refers to ensuring that the planned timelines for procurement process steps are strictly adhered to. In this view, ascertaining the performance of public procurement processes, process timeliness cannot be ignored or rated low.

The PPA has attempted to ensure timeliness in Ghanaian procurement processes. Consequently, tools, manuals, and techniques have been developed to aid procurement timeliness. One of these is the [33]. **In this manual, time limits are proposed for the various procurement methods.**

There are various procurement methods applicable in Ghana, but the most preferred and widely used method of procurement by public entities for the procurement of works is competitive tendering. This is because they encourage maximum competition. Hence, there is a focus on the competitive tendering method of procurement in this study. Competitive tendering is the tendering process where an advertisement is placed in the dailies by the client inviting prospective bidders to bid after depositing a non-refundable amount for the tender document in which all necessary information about the proposed project and tendering process can be found [4].

The competitive tendering process flow adopted for this study spanned from preparation of tender documents; advertisement; receipt of bids; opening of bids; evaluation of bids; approval for award; and award of contract. It is necessary to note that, the competitive tendering process used in this study followed the process outlined in Ref. [34]. It has been argued that competitive tendering is characterized by poor performance due to delays [11].

### 2.5 Performance of competitive tendering in Ghana

Public procurement all over the world has experienced undue delays [10]. These delays have damaged the image of entities and wasted scarce public resources. Noteworthy are the delays between bid solicitation and contract award also known as the tendering process. Table 3 presents the stipulated timelines for the international competitive tendering method in Ghana.

**Table 3:** Prescribed timelines for competitive tendering in procurement manuals in Ghana.

Process steps	International competitive tender	
	Timelines in weeks for lower limits	Timelines in weeks for upper limits
Preparation of Tender documents	2	2
Prior review/approval of Tender documents	1	2
Advertisement	6	8
Tender closing/opening	–	–
Tender evaluation and report submission	2	4
Post review/no objection	1	2
Contract award	1	2
**Total**	**13**	**20**
**Mean timelines**	**16.5**	

Source: Ghana Public Procurement Authority (2006).

The stipulated timelines suggest an awareness of the need for a timely process by the PPA. It is therefore expected that the assessment of public procurement processes by the PPA would include ascertaining the compliance of entities with these timelines. However, these time limits were not featured in the performance measurement criteria by the PPA. There is therefore a need to design an index that can measure the timeliness of public procurement processes. A measure that can compare planned timelines to actual timelines to ascertain the existence or extent of delays. The newly proposed procurement delay index based on the Six Sigma metric fills this gap.

## 3 Materials and methods

The epistemological position of this research is pragmatic. The reason is that the research focuses on making a difference in organizational practice [35] by exploring the possibility of adding the *PDindex* to the assessment method of procurement processes to gradually expose procurement practitioners to quality tools. This was achieved by applying the *PDindex* to competitive tendering processes within a real-life context. Thus, the research started with solving a problem within a specific context and informing future practice as a contribution to knowledge to provide a practical meaning to knowledge. Therefore, the research design and strategy were based on the problem to be solved and the research objectives.

Ontologically, this research strives to reconcile objective and subjective positions by reconciling facts and values [35] through the review of documentary evidence and the application of statistical tools. Thus, it reviewed the literature to identify theories on the application of Six Sigma to procurement processes. It also analyzed documentary evidence using both quantitative and qualitative techniques such as frequencies and content analysis. This resulted in the application of the newly designed *PDindex* within a real-life context which also quantified qualitative data to establish results.

This research modifies existing theory and applies it within a new contest to validate it making its approach abductive [35]. The reason is that the research builds on existing metrics and applies them to the competitive tendering process to explore the possibility of improving performance measurement of procurement processes. Hence, the design for this research is exploratory. A case study strategy was adopted for this research. The type of case study adopted was a single case study. This was because an empirical investigation within a real-life context was required to explore the application of *PDindex* to the procurement process [36].

This research employed the merged approach under the mixed method. Thus, qualitative data was collected and analyzed qualitatively as well as quantitatively at certain stages of the research using frequencies of specific events and graphs. To effectively apply the *PDindex* some of the qualitative data had to be quantitised [35] due to the nature of the Six Sigma tools. This research assumed a longitudinal time horizon as it reviewed documentary evidence from 2013 to 2015 to determine a trend. This trend assisted in defining the problem within the case studied.

Data was collected by selecting a benchmark entity for the case study. A comprehensive study of the entity's records on the competitive tendering processes from 2013 to 2015 was conducted. This was because the procurement processes used for the 2014 assessment spanned from 2013 to 2015. The study was based on processes of projects adopting international competitive tendering procedures. The competitive tendering records of the entity were reviewed to determine the number of activities and number of defects. This was done by screening each process within the entity to determine whether they met the planned timelines. The findings formed the basis for computing the *PDindex*. This was done by modifying the method applied by Ref. [13] to compute the DPMO of the process using equations (1), (2), (3), (4), (5)).

(1) $$\text{Defects}\;\text{Per}\;\text{Unit}\;\left(\text{DPU}\right)=\frac{\text{Defects}}{\text{Activities}}$$

(2) DefectsPerOpportunity=(DPO)

(3) DefectsPerMillionOpportunity=(DPMO)

(4) $$\text{DPO}=\frac{\text{DPU}}{\text{Average}\;\text{Opportunities}\;\text{for}\;\text{Error}}$$

(5) $$\text{DPMO}=\text{DPO}\times 10^{6}$$

The DPMO formed the basis for establishing the sigma level through interpolation or graph. After developing the index, it was validated by applying it to real-life procurement processes within a benchmark entity in Ghana.

## 4 Results

### 4.1 Development of procurement delay index

Sigma level is applied to normally distributed data whereas DPMO is more appropriate for data that is not normally distributed [37,20]. Thus, traditionally sigma level metric is preferred to DPMO because of the ease of calculation as a large percentage of social science data is normally distributed. However, the greatest challenge with the sigma level metric is the prevalence of misinterpretation of its value as compared to more objective DPMO [37]. Therefore, for objective performance evaluation, the reverse transformation of the sigma level into DPMO is ideal [37]. The new PD*index* has thus been developed by combining the concept of DPMO and sigma level metric to avert any possible misinterpretation of sigma level performance as it is relatively new in procurement.

It is however necessary to note that the corresponding sigma level and DPMO referred to in this paper is the long-term sigma level as it is the industry's preferred choice [37]. This takes into consideration the 1.5 process average shift due to normal causes of variation [18]. PD*index* adopts the traditional computation formula for DPMO after which it uses bell shaped curve to determine the sigma level. Interpolation can equally be used to determine the sigma level.

To enhance the uniqueness and acceptability of the PD*index* by procurement professionals, the traditional quality terms have been revised to reflect specific procurement delay terms. This also informed the decision to think discretely instead of continuously in its application. Below are the specific revisions:

1. Defect per unit (DPU) is equivalent to Delay per unit *(DlPU)*
2. Defect per opportunity (DPO) is equivalent to Delay Per Procurement Process *(DlPP)*
3. Defect per million opportunities is equivalent to Delay Per Million Procurement Processes (*DlPMPP)*
4. Defects are equivalent to Delays (not meeting planned timelines)

## 5 Activities are equivalent to procurement processes

Thus, after computing the *DlPMPP*, either the bell-shaped curve or simple interpolation is used to derive the PD*index*. The PD*index* provides a specific value representing procurement process performance concerning meeting planned timelines.

Focusing on procurement process timeliness, there is a need to ascertain the level of process delay. This forms the basis for determining the timeliness of an entity's procurement process. It is therefore necessary to identify the following:

1. The minimum and maximum allowable timelines for a particular procurement method are established by either the PPA or the entity in its procurement plan. This will assist in computing the average planned period for a procurement process.
2. The actual average process duration for all procurement processes using the method in 1 above within a specified period. This can be extracted from the updated procurement plan.

Having identified the above, the delay per million procurement process (*DlPMPP*) is computed using equations (6), (7), (8)).

(6) $$Delay\;Per\;Unit\;\left(DlPU\right)=\frac{Number\;of\;procurement\;processes\;that\;delay}{Total\;number\;of\;procurement\;processes}$$

(7) $$DlPP=\frac{DlPU}{1}$$

(8) $$DlPMPP=DlPP\times 10^{6}$$

where:

*DlPP* is Delay Per Procurement Process.

*DlPMPP* is Delay Per Million Procurement Processes.

Then, using the normal distribution curve or simple interpolation, the *DlPMPP* is used to establish the *PDindex*. Further, using the allowable 3σ as an acceptable rate of delay [23] representing 99.7% of procurement processes meeting planned timelines, a rating scale to rank the performance of procurement processes concerning process cycle time has been proposed in Table 4.

**Table 4:** Relationship between PD*index* and rating for procurement process timeliness.

Procurement Delay Index (σ)	Rating of procurement process timeliness	Equivalence in days
2.6σ to 3.0σ	Excellent procurement process timeliness (excellent)	91–100
2.1σ to 2.5σ	Very good procurement process timeliness (matured)	101–110
1.6σ to 2.0σ	Good procurement process timeliness (maturing)	111–120
1.1σ to 1.5σ	Average procurement process timeliness (conforming 1: average)	121–130
0.6σ to 1.0σ	Poor procurement process timeliness (conforming 2: below average)	131–140
0.1σ to 0.5σ	Untimely procurement process (non-conforming)	141–150
0 σ	Highly untimely procurement process (non-conforming)	Beyond 151

It is necessary to note that the PD*index* must be rounded to the nearest decimal place to rate the process. Table 4 also proposes equivalent *PDindex* to days following the prescribed maximum and minimum timelines presented in Table 3. The rating has been designed to follow the terminologies used by the PPA in Appendix 2. Entities can monitor their performance in days and relate it to the equivalent *PDindex* to ascertain the timeliness of their procurement processes.

To compute the traditional DPMO, it is necessary to identify the activities, defects, and opportunities for error. Additionally, the successful application of Six Sigma in service requires the establishment of some critical success factors including top management involvement and commitment; a proper understanding of the process; and keeping up-to-date records [38]. These parameters and critical success factors are identified in the procurement language to apply PD*index* to public procurement processes. These have been described as prerequisite condition for the application of PD*index*. The following conditions must be met by the public entity.

Condition 1 Top management involvement and commitment.

Condition 2 The entity prepares its annual procurement plan according to the guidelines in the [25,26,39]. Plans must show the planned timelines for various procurement process steps.

Condition 3 The entity updates its procurement plan quarterly according to the [25,26,39]. Plans must be updated to show the actual timelines of the various procurement process steps that were carried out.

Condition 4 Proper record keeping where evidence of timelines for various process steps can be found.

Having met the above conditions, the following steps must be followed to compute the PD*index*.

Step 1 Establish the assessment period in consultation with top management.

Step 2 Decide on the procurement process to be assessed in consultation with top management. For example, goods, work, or services.

Step 3 Decide on the procurement method to be assessed in consultation with management. For example, national competitive tendering, international competitive tendering, or price quotation.

Step 4 From the procurement plan, select the procurement processes that fall within the criteria in steps 1-3 above. This will provide the *total number of procurement processes*.

Step 5 From the updated procurement plan, extract the planned and actual timelines for each procurement process of the processes established in 4 above. Compute the average allowable timeline for the entire procurement process by summing up the upper and lower specification limits and dividing by two. Then compute the same for the actual timelines of the individual processes. Further, compare the average planned timeline to the average actual timeline to determine the processes that were completed within the planned timelines and those that were not. The processes that were not completed within the planned timelines are the ‘number of procurement process delays’.

Step 6 Using equations (1), (2), (3)) compute the DlPMPP of the procurement process.

Step 7 Using the normal distribution curve and the DlPMPP determine PDindex. This is achieved by plotting the normal distribution curve using the values in Table 1 with DlPMPP on the vertical (y-axis) and the sigma on the horizontal (x-axis). The relationship between the sigma level and DlPMPP is achieved by adopting the values in Table 1 but in procurement terms. This follows the relationship between the traditional sigma level and DPMO. Then the value for DlPMPP determined in Step 6 is established on the y-axis and its correlating PDindex (sigma level) is determined on the x-axis using the normal distribution curve as the reference point.

Step 8 Using Table 4 determine the rating of timeliness of the procurement processes and its corresponding timeline in days.

### 5.1 Application of PDindex to public procurement processes

A case study was used to demonstrate the practical application of PD*index* by applying it to measure the amount of delay or level of timeliness in the competitive tendering process (CTP). The focus was on CTP because at the time of the study, the entire procurement process of the case study entity was incomplete. Available data was on the tendering process. The entity used international competitive tendering for the procurement of their “works” hence the decision to select that procurement method. This entity was one of the benchmark entities in the 2014 assessment exercise by the PPA. It was selected for the study because it was one of the best candidates for the study as it fully met conditions 1 to 4 for applying the PD*index* discussed above. Procurement records from 2013 to 2015 for international competitive tendering processes were used for this research. First determining whether the entity meets the conditions for the application of the PD*index* (Stage 1) and second, the step-by-step application of the PD*index* (Stage 2).

**Fig. 1:**
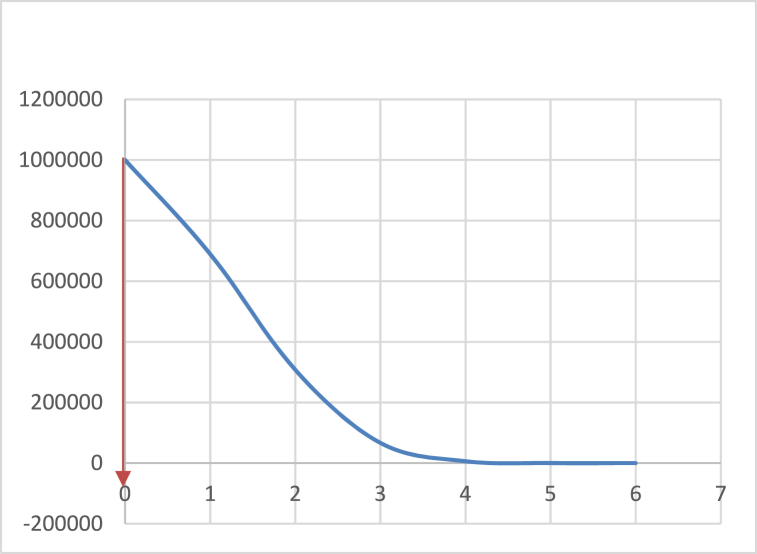
Caption: Sigma Level for Case study Fig. 1. A graph depicting the sigma level of the case study where DPMO is on the Y-axis and process sigma is on the X-axis. Thus, a DPMO of 1,000,000 leads to a sigma level of 0 σ for the case study.

#### 5.1.1 Stage 1

Condition 1: Permission was granted by the top management of the entity after researchers submitted a written request to conduct the study. Top management agreed for researchers to review all their procurement records within the agreed period.

Condition 2: The annual procurement plans for the entity within the stipulated period were inspected to ascertain whether they followed the guidelines of the acts and procurement manuals.

Condition 3: The procurement plan showed the planned and actual timelines for each process step.

Condition 4: There was evidence to support the timelines in the procurement plans as up-do-date records were kept on procurement.

Having met all four conditions above, the practical application of PD*index* is presented in the next section.

#### 5.1.2 Stage 2

Step 1: In consultation with the management of the entity, the period under study was agreed to be 2013 to 2015. Procurement records from 2013 to 2015 for international competitive tendering processes were reviewed.

Step 2: In consultation with management, the procurement processes for ‘works’ were selected for the study. This was because the procurement processes for works had up-to-date records. These were also the processes that recorded the highest delays.

Step 3: In consultation with the management, the processes using the international competitive tendering method were selected. This was the only method used to procure works contracts between 2013 and 2015.

Step 4: Documentary analysis was done to compare the average planned timelines to actual timelines. Table 5 shows the details of process performance over time. The actual timelines for the various process steps for each ICT process within the stipulated time were determined. Then mean timeline for all the 7 processes under study was computed as shown in Table 5.

**Table 5:** Performance of competitive tendering processes in relation to time.

Number of Competitive Tendering Processes	Procurement Method	Average Planned Timeline Per Process (Weeks)	Average Actual Timeline Per Process (Weeks)	Average Delay Per Process (Weeks)	Average Delay Per Process (Days)	Number of Timely Processes
**7**	**ICT**	**33**	**81**	**48**	**336**	0
CTP1	ICT	33	74	41	287	–
CTP2	ICT	33	74	41	287	–
CTP3	ICT	33	74	41	287	–
CTP4	ICT	33	88	55	385	–
CTP5	ICT	33	88	55	385	–
CTP6	ICT	33	84	51	357	–
CTP7	ICT	33	84	51	357	–

Step 5: From Table 5, the ‘number of procurement process delay’ is seven (7).

Step 6: Delay Per Million Procurement Processes in Case is presented in equations (9), (10), (11)).

(9) DelayPerUnit(DlPU)=7∕7=1

(10) DlPP=1∕1=1

(11) $$DlPMPP=1\times 10^{6}=1,000,000$$

From the above computations, the case recorded a *DlPMPP* of 1,000,000. This means that all competitive tendering processes undertaken by the case failed to meet their planned deadlines.

Step 7: The above information was used to determine the PD*index* of the competitive tendering process as shown in Fig. 1 and its corresponding number of days.

Step 8: From Table 4, a PD*index* of 0σ translates into a highly untimely procurement process (non - conforming) with its corresponding duration of 336 days which is beyond 151 days.

## 6 Discussion of results

All the competitive tendering processes in the case study failed to meet their planned deadlines and hence were delayed as can be seen in Table 5. When these figures are compared with the stipulated average timelines of 16.5 weeks (115.5 days) from the [39] in Table 3, the situation becomes worse. Note that this entity was ranked excellent based on their performance between 2013 and 2015 but had a *PDindex* of 0 σ. The existence of delays in this case confirms the assertion by Ref. [10] that delays persist in public procurement processes. Considering the correlation between time and cost, and the effects of delays on construction projects [40] it is not likely that this omission by the PPA was deliberate.

Even though three sub-criteria of the 2014 assessment exercise touched on the timeliness of the activities, there was no specific criterion to solely compare the planned timelines of the various process steps to the actual timelines. It can be concluded that the existing assessment method allocated less than 2% of its scores to ascertain the rate of delays. This confirms the findings of [11] that the timeliness of tendering processes in Ghana has been ignored in determining the performance of procurement processes. Even though the entity had up-to-date records on the planned and actual timelines of the various process steps, the PPA underrated the importance of accurately ascertaining delays in its performance assessment of the entities leading to the ranking of this entity as excellent. The reason is that any excellent ranked entity according to Appendix 2 could lose up to 20% of the total score and still be ranked excellent. This ranking approach can be considered flawed and does not reflect reality. It supports the assertion by Ref. [41] that government e-procurement systems are generating tender-level process event data which are not being analyzed much.

A tangible justification for this flawed assessment of public entities is the Ghanaian laxed approach to time as suggested by The Blog for Culture Vultures in 2013. This lax attitude is engraved in Ghanaian culture to the extent that process timelines, even though seen as an important performance measure, were severely underrated by the PPA in public procurement processes assessment. This finding supports that of [27] which concluded that one of the areas lacking measurement include delivery cycle time. Even though it is acknowledged that establishing a measurement system for process timeliness is hard work, the advantages outweigh the costs and efforts involved in the implementation of such a system. Further, the application of the rating scale proposed in Table 4 reduces the assessment workload. There is therefore the need to add the *PDindex* to the existing procurement performance assessment systems to solely measure the timeliness of procurement processes. This meets the requirement in Section 21 of [25] where public entities are expected to prepare their annual procurement plans and quarterly updates.

*PDindex* provides a clear, consistent, and non-conflicting rate of delay. This finding has confirmed the findings in the literature where Six Sigma was identified as a performance improvement strategy applicable to procurement processes [7,42]. Thus, *PDindex* can be added to the balanced scorecard and capability maturity model originally used in assessing procurement performance. Doing so will ensure that entities are ranked excellent only when their PD*index* is 3. This will address the flaws in the existing performance measurement criteria and enhance the accuracy of the measurement system.

Critically analyzing Table 3, Table 5 and it can be seen that the average planned timelines for international competitive tendering processes are 16.5 weeks (115.5 days) and by the entity 33 weeks (231 days) respectively. Thus, entity's planned timeline was twice the stipulated timelines in Ref. [33]. A possible reason for this could be that the timelines stipulated in Ref. [33] are unrealistic and hence the entity stipulates its timelines. This suspicion is further strengthened by the actual results on the actual timelines observed with the minimum average actual time being 74 weeks (518 days). Even though the stipulated timelines in Ref. [33] reflect the minimum allowable timelines, the difference between the timelines in the entity's procurement plan leaves more questions that require answers.

There is therefore a need for the PPA to reassess the stipulated timelines prescribed in the [33] by applying tools that can realistically predict the duration of process steps. This is long overdue because the [25] was released in 2003 and the [33] was released in 2006. Following the release of these two documents, the [25] was amended in 2014 with [26]. Further, in 2022 the [43] was released. Despite these amendments, revisions, and additions, there have not been any revisions on [33], the only document that stipulates timelines for specific procurement activities.

## 7 Conclusion and future research

In today's era of economic hardships, the need to address procurement process delays cannot be over-emphasized due to its corresponding cost implications. In this paper, it has been established that the assessment criteria for public procurement performance in Ghana do not accurately assess procurement process delays. Thus, the paper sought to highlight the assessment of delays in public procurement processes by deriving the level of procurement delays using the *PDindex* with sigma (σ) as its unit of measurement.

*PDindex* has been developed to serve as an assessment tool for procurement process delays that can complement the existing assessment criteria. The index also determines the timeliness of the process by rating them and providing the corresponding duration in days. An example has been used to illustrate the practicality of the proposed measurement scheme. *PDindex* serves as a standard for determining whether procurement processes meet their stipulated timelines. It also provides a less expensive way of implementing Six Sigma in procurement processes and creates awareness of Six Sigma in procurement.

A PD*index* of 3σ correlating with a period of between 91 and 100 days has been recommended to be the acceptable limit for excellent procurement process performance in terms of delay. The results demonstrate that the PD*index* could be of considerable assistance in the assessment of procurement process delays. To this end, based on the application perspective, the proposed PD*index* can further be applied in the future to accurately establish benchmarks or excellent performing entities in public procurement processes. Additionally, timelines prescribed in Ref. [33] should be reassessed to ascertain their accuracy and workability. There may be a need to correspondingly revise the rating scale for PD*index* if subsequent revisions are made to the stipulated timelines in Ref. [33].

## Data availability statement

1. The following data are openly available in a public repository that does not issue DOIs.
a 2014 Maturity Table is openly available at the Ghana Public Procurement website at https://ppa.gov.gh
2. Further details of the performance criteria that formed the basis for the 2014 maturity table have been attached as supplementary material for review.
3. Due to the nature of this research, participants of this study did not agree for the identity of the case study be shared publicly. Also, information was extracted mostly from hard-copy documents. Thus, supporting data beyond what is in the paper is not available from the case study.

## CRediT authorship contribution statement

**Jemima A. Ottou:** Writing – review & editing, Writing – original draft, Funding acquisition, Formal analysis, Data curation, Conceptualization. **Bernard K. Baiden:** Writing – review & editing, Supervision, Investigation. **Gabriel Nani:** Writing – review & editing, Supervision, Methodology. **Martin Morgan Tuuli:** Writing – review & editing, Validation, Methodology.

## Declaration of competing interest

The authors declare that they have no known competing financial interests or personal relationships that could have appeared to influence the work reported in this paper.

## Footnotes

Appendix A Supplementary data to this article can be found online at https://doi.org/10.1016/j.heliyon.2024.e25672.
