# Supplementary material for: Enhancing performance measurement of public procurement processes through the application of procurement delay index
Source: Heliyon. 2024 Feb 6;10(4):e25672. doi: 10.1016/j.heliyon.2024.e25672 (PMC10877244; doi:10.1016/j.heliyon.2024.e25672)
Supplement: Multimedia component 2 [file mmc2.docx]

| Stages | Stage 1 | Stage 2 | Stage 3 | Stage 4 | Stage 5 |
| --- | --- | --- | --- | --- | --- |
| CHARACTERISTICS | NON-CONFORMING | CONFORMING | MATURING | MATURED | EXCELLENCE |
| PROCUREMENT STRUCTURES | Prescribed structures not in place. No Procurement Unit | Structures and Procurement Unit in place but Procurement Unit is not resourced or functional | All structures are in place including a Procurement Unit which is resourced but partially functional | All structures are in place including procurement Unit which is resourced and functional | All structures are in place including procurement Unit which is well resourced and functional |
| PROCUREMENT STAFF | No qualified or trained Procurement staff | Procurement Unit has no qualified procurement professionals. Staff in charge of procurement however have some training in procurement | Has at least one qualified procurement professional who is the Head of the Procurement Unit | All procurement staff have professional qualification but not fully involved in all categories of procurement. | All procurement staff have professional qualification and fully involved in all categories of procurement. |
| PROCUREMENT PLANNING | No procurement plans are prepared | No procurement plans are prepared | Procurement plans prepared but not posted on PPA Website | Procurement Plan prepared and posted on PPA’s Website. Procurement Plan is not fully updated with information on “Actuals” | Procurement plan prepared and posted on PPA’s Website. Procurement Plan is updated with information on “Actuals” |
| PROCUREMENT PERFORMANCE | | | | | |
| Total Weighted Score | 0-20 | 21-40 | 41-60 | 61-80 | 80+ |
| Performance Rating | Poor | Fair | Good | Very Good | Excellent |
| Areas of Improvement | 41 - 59 | 26 - 40 | 11-25 | 1-10 | 0 |
